# Prognostic Biomarkers on a Competitive Endogenous RNA Network Reveals Overall Survival in Triple-Negative Breast Cancer

**DOI:** 10.3389/fonc.2021.681946

**Published:** 2021-06-11

**Authors:** Wenxing Qin, Feng Qi, Jia Li, Ping Li, Yuan-Sheng Zang

**Affiliations:** ^1^ Department of Oncology, Changzheng Hospital, Naval Medical University, Shanghai, China; ^2^ Liver Cancer Institute, Zhongshan Hospital, Fudan University, Shanghai, China; ^3^ Department of Gastroenterology, Changhai Hospital, Naval Medical University, Shanghai, China

**Keywords:** competitive endogenous RNA, triple-negative breast cancer, prognostic model, differentially expressed genes, nomogram

## Abstract

The objective of this study was to construct a competitive endogenous RNA (ceRNA) regulatory network using differentially expressed long noncoding RNAs (lncRNAs), microRNAs (miRNAs), and mRNAs in patients with triple-negative breast cancer (TNBC) and to construct a prognostic model for predicting overall survival (OS) in patients with TNBC. Differentially expressed lncRNAs, miRNAs, and mRNAs in TNBC patients from the TCGA and Metabric databases were examined. A prognostic model based on prognostic scores (PSs) was established for predicting OS in TNBC patients, and the performance of the model was assessed by a recipient that operated on a distinctive curve. A total of 874 differentially expressed RNAs (DERs) were screened, among which 6 lncRNAs, 295 miRNAs and 573 mRNAs were utilized to construct targeted and coexpression ceRNA regulatory networks. Eight differentially expressed genes (DEGs) associated with survival prognosis, DBX2, MYH7, TARDBP, POU4F1, ABCB11, LHFPL5, TRHDE and TIMP4, were identified by multivariate Cox regression and then used to establish a prognostic model. Our study shows that the ceRNA network has a critical role in maintaining the aggressiveness of TNBC and provides comprehensive molecular-level insight for predicting individual mortality hazards for TNBC patients. Our data suggest that these prognostic mRNAs from the ceRNA network are promising therapeutic targets for clinical intervention.

## Background

Breast cancer is the most frequently diagnosed cancer and the second most common cause of cancer mortality in women worldwide (1). TNBC, as an aggressive subtype, accounts for 12-18% of all breast cancers (2). Because TNBC patients lack the oestrogen receptor (ER), progesterone receptor (PR) and HER2 receptors, they are not suitable for hormone or anti-HER2 therapy. Targeted therapies cannot significantly improve the survival rate of TNBC patients, and chemotherapy is still the standard treatment. Consequently, exploring the molecular biological mechanism affecting the prognosis of patients with TNBC and identifying reliable prognostic markers are very valuable for accelerating individual therapies and improving clinical prognoses.

Over the past decade, substantial efforts have been made to classify TNBC into distinct clinical and molecular subtypes to guide treatment decisions. The characterization of genomic, proteomic, epigenomic and microenvironmental changes has expanded our understanding of TNBC. High levels of somatic mutations, frequent TP53 mutations (83%) and complex aneuploidy rearrangement (80%) have been found in TNBC patients *via* deep sequencing studies (3), multiregion sequencing analyses (4) and single-cell sequencing research (5), revealing extensive intratumoural heterogeneity (ITH). However, the molecular mechanism driving TNBC relapse has not been fully elucidated.

The ceRNA hypothesis involves a specific molecular biological regulatory mechanism of posttranscriptional regulation (6). Studies have explored the mechanism of ceRNA biological regulation in TNBC patients. Yuan et al. (7) proposed a ceRNA crosstalk network in triple-negative breast cancer *via* integrative analysis of lncRNAs and miRNAs with coding RNAs. Song et al. (8) established a ceRNA topology network with five specific RNAs: hsa-miR-133a-3p, hsa-miR-1-3p, TRIML2, TERT and PHBP4. However, due to their complex formulas, unclear results, lack of external validation and other reasons, these prediction models do not predict prognostic effects very well. A prognostic model with a simple formula, easily interpretable results and strong external repeatability are very important for optimizing individualized treatment. Therefore, the purpose of this study was to develop and validate a nomogram for determining OS prognoses in patients with TNBC based on gene expression data confirmed by the ceRNA regulatory network and to screen potential therapeutic agents among existing small-molecule inhibitors.

## Methods

### Expression Profile Data Screening

The breast cancer expression data (including mRNAs, lncRNAs and miRNAs) were downloaded from The Cancer Genome Atlas (TCGA) database (https://gdc-portal.nci.nih.gov/), and the Illumina HiSeq 2000 RNA platform was used for sequencing. The downloaded data was TCGA TNBC RNA-seq level 3 with a normalized log (FPKM+ 1,2) that can be used for direct analysis. After comparing the clinical information of the downloaded samples (**Table S1**), the following breast cancer samples were retained: patients with known expression levels of mRNAs, lncRNAs and miRNAs; patients negative for ER, PR and HER-2; patients with complete pathological staging and follow-up data; and enrolled patients with TNBC primary disease and no other malignant tumours. A total of 102 samples and 63 control samples were obtained, and these samples were used as the training group. Moreover, the RNA-seq breast cancer data were downloaded from the Molecular Taxonomy of Breast Cancer International Consortium (Metabric) database (http://molonc.bccrc.ca/) and included a total of 1904 breast cancer patients with corresponding clinical information. Based on the above screening criteria, we selected 298 TNBC samples, and this data set was used as the validation group. All of the data sets utilized in this study were from public databases. Ethical approval was not required because researchers are allowed download and analyse data from these public databases for scientific purposes.

### Data Preprocessing and Screening of Differential Genes

LncRNAs, miRNAs and mRNAs detected in the TCGA samples were annotated. Then, the R language package Limma (version 3.34.0, https://bioconductor.org/packages/release/bioc/html/limma.html) (9) was used to screen for differentially expressed RNAs (DERs) between the TNBC and control (CTRL) groups. The screening threshold was an false discovery rate (FDR) < 0.05 and a |log2FC| > 0.5. The R language package pheatmap (version 1.0.8, https://cran.r-project.org/package=pheatmap) (10) was used to perform bidimensional hierarchical clustering (11, 12) analysis based on the Euclidean distances (13) of the expression levels of the selected DERs, which are displayed in the heatmap.

### Construction of a Targeted Regulatory ceRNA Network

We analysed the relationships of differentially expressed (DE) lncRNAs and miRNAs from the DIANA-LncBase database (14) (https://diana.e-ce.uth.gr/lncbasev3) and retained only the pairs with opposite expression trends. The StarBase database (15) (version 2.0, http://starbase.sysu.edu.cn/) was used to search the differentially expressed genes (DEGs) correlated with DEmiRNA regulation, and only the negatively correlated miRNA and mRNA pairs were retained. Based on the above results, a ceRNA regulatory network composed of DElncRNAs, DEmiRNAs, and DEmRNAs was constructed, and the network was visualized with Cytoscape (Version 3.6.1) (https://cytoscape.org/) (16). The regulatory network of ceRNA coexpression was used to calculate the Pearson correlation coefficients (PCCs) between the expression levels of DElncRNAs *vs*. DEmiRNAs, DElncRNAs *vs*. DEmRNAs and DEmiRNAs *vs* DEmRNAs by using the Cor function in R3.6.1 software (http://77.66.12.57/R-help/cor.test.html), and the pairs with significant correlation (*P* < 0.05) were selected. Finally, The Database for Annotation, Visualization and Integrated Discovery (DAVID) (version 6.8) (17, 18) (http://metascape.org/) was used to conduct Gene Ontology (GO) biological process and Kyoto Encyclopedia of Genes and Genomes (KEGG) pathway enrichment analyses of the DEGs in the ceRNA regulatory network, and *P* < 0.05 was selected as the threshold for determining significant enrichment.

### Screening of Prognostic-Related Genes

In the TCGA training set, based on the ceRNA regulatory network and the prognostic information obtained from the samples, univariate Cox analysis was used to screen mRNAs correlated with significant overall survival (OS) with R software (survival pack, version 2.41, http://bioconductor.org/packages/survivalr/). Multivariate Cox regression analysis was used to screen for mRNAs with significant independent prognostic correlation using the survival package (version 2.41-1) (19) of R3.6.1. A log-rank *P* value < 0.05 was selected as the threshold for determining significant prognostic mRNAs.

### Establishment of the Prognostic Risk Prediction Model

Based on multifactor Cox regression analysis of mRNAs and their expression levels in the TCGA, we constructed the following prognostic score (PS) model:

$Prognostic\;score\;\left(PS\right)\;=\;\sum Coef_{mRNAs}\times \;Exp_{mRNAs}$

Coef _mRNAs_ represents the prognostic coefficients of mRNAs in the multivariate Cox regression analysis, and Exp _mRNAs_ represents the expression levels of mRNAs in the TCGA dataset.

### Statistical Analysis

The t-test or Mann‐Whitney U test was used for comparison, and continuous variables are expressed as the mean ± standard deviation. The χ2 test or Fisher’s exact test was used to compare the categorical variables. The prognostic factors were computed using the Cox proportional hazards model, where HR was the 95% confidence interval. OS was defined as the time from the date of diagnosis to the date of last follow-up or death and was analysed by the log-rank test. Nomograms and ROC curves were used to evaluate the predictive performance of the prognostic model. All statistical analyses were computed using SPSS version 22.0 (IBM SPSS Statistics, Chicago, IL, US), and R software (version 3.5.2) with the following packages: “Limma”, “pheatmap”, “survival”, “rmda”, “cor”, “GOplot”, “ROC”, “rms”. *P* < 0.05 was considered statistically significant.

## Results

### Data Preprocessing and Screening for Significantly DERs

**Figure 1:**
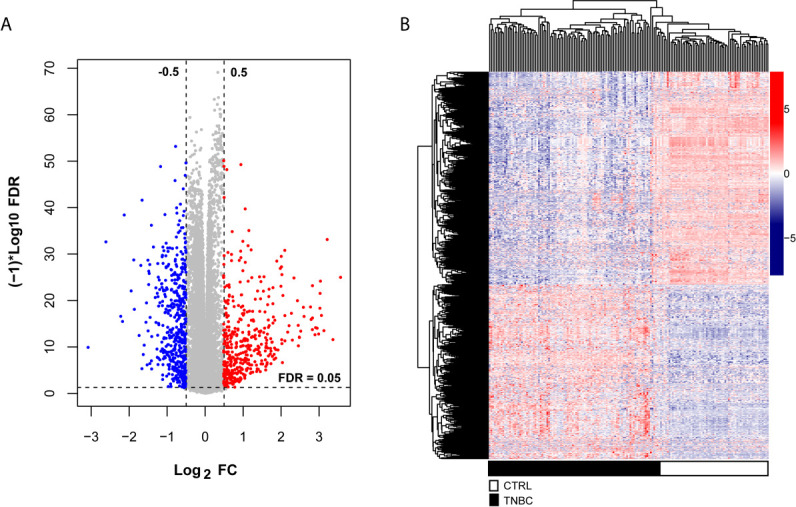
Differentially expressed RNAs, including lncRNAs, miRNAs and mRNAs, from the TCGA dataset. **(A)** The volcano map was constructed. The blue and red dots indicate significantly downregulated and upregulated differentially expressed RNAs, respectively. The horizontal dotted line indicates an FDR < 0.05, and the two vertical dashed lines indicate a | log2FC | > 0.5. **(B)** Bidirectional hierarchical clustering heat map based on the levels of differentially expressed RNAs; the black and white sample bars represent TNBC and control peritumoral tissue patients, respectively.

To explore the significant prognostic correlate factors for triple-negative breast cancer, we selected the patients with triple-negative breast cancer from TCGA and Metabric data sets. There were 102 TNBC patients in the training group and 298 TNBC patients in the validation group. The clinical and demographic characteristics of all patients with TNBC are summarized in **Table 1**. According to the platform annotation information provided in the downloaded data, 14,000 mRNAs, 1778 lncRNAs and 2222 miRNAs were annotated in the data set. A total of 874 DERs were screened, among which 6 lncRNAs, 295 miRNAs and 573 mRNAs met the screening threshold criteria (**Table S2**). The inspection volcano distribution map is shown in **Figure 1A**. The bidirectional hierarchical clustering heat map based on DER expression is presented in **Figure 1B**. The above DERs were identified as candidate prognosis factors.

**Table 1:** Clinical characteristics of TNBC patients in the training and validation groups.

Clinical characteristics	Training group	Validation group	P value
	N = 102	N = 298	
Age (years, mean ± SD)	57.01 ± 11.75	55.66 ± 13.76	3.38E-01
Pathological M (M0/M1/-)	85/1/16	NA	NA
Pathological N (N0/N1/N2/N3/-)	65/23/11/3	NA	NA
Pathological T (T1/T2/T3/T4)	23/64/11/4	NA	NA
Pathologic stage (I/II/III/IV/-)	16/65/17/1/3	62/130/25/0/81	**2.48E-02**
Radiotherapy (Yes/No/-)	53/40/9	214/84	**1.04E-02**
Recurrence (Yes/No/-)	15/78/9	NA	NA
Death (Yes/No)	16/86	161/137	**3.50E-12**
Overall survival time (months, mean ± SD)	38.95 ± 31.34	113.42 ± 83.37	**2.62E-12**

TNBC, triple-negative breast cancer; NA, not applicable. A P value < 0.05 was considered to be statistically significant. The bold values represent statistically significant.

### Construction of the ceRNA Networks and Functional Analysis

**Figure 2:**
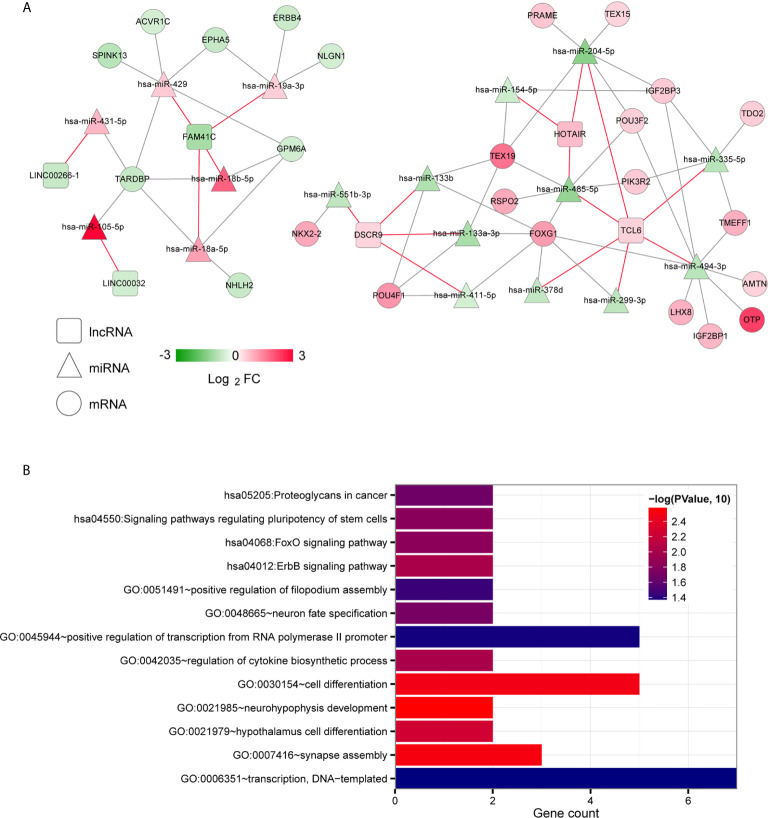
Targeted ceRNA regulatory network. **(A)** The mutual targeting relationships among lncRNAs, miRNAs and mRNAs. The colour change from green to red indicates a change from low to high. The red and grey lines indicate the DElncRNA-DEmiRNA associations and the DEmiRNA-DEmRNA regulatory associations, respectively. **(B)** Bar diagram of the GO biological processes and KEGG signalling pathways significantly correlated with mRNAs in the ceRNA network. The horizontal axis shows the number of genes. The vertical axis shows the entry names. Entries closer to a red colour are more significant.

To further explore the biological functions and involvement in signaling pathways of DERs, we constructed the ceRNA networks from DERs including lncRNAs, miRNAs and mRNAs. The ceRNA regulatory networks were constructed in two ways: targeted and coexpression regulation. From the binding relationships between DElncRNAs and DEmiRNAs screened in the above step based on the DIANA-LncBasev2 database, we retained only the pairs exhibiting opposite expression trends, yielding total 19 pairs (**Table S3**). Then, we searched the target genes regulated by DEmiRNAs by using the StarBase database and matched the significant DEGs obtained in the first step. Moreover, only pairs with negatively correlated miRNA and mRNA expression levels were retained, yielding 50 total pairs. A targeted ceRNA network composed of lncRNAs, miRNAs, and mRNAs was constructed after synthesizing and collating DElncRNAs, DEmiRNAs, and DEmiRNA-DEmRNAs (**Figure 2A**). Finally, enrichment annotation analyses of GO biological processes and KEGG signalling pathways associated with the mRNAs involved in the ceRNA regulatory network were carried out based on DAVID. We identified 9 significantly related GO biological processes, including neurohypophysis development, synapse assembly, cell differentiation, hypothalamus cell differentiation, regulation of cytokine biosynthetic process, neuron fate specification, positive regulation of filopodium assembly, positive regulation of transcription from RNA polymerase II promoter and transcription, and 4 KEGG signalling pathways, including the ErbB signalling pathway, FoxO signalling pathway, and signalling pathways regulating stem cell pluripotency and proteoglycans in cancer (**Figure 2B**).

**Figure 3:**
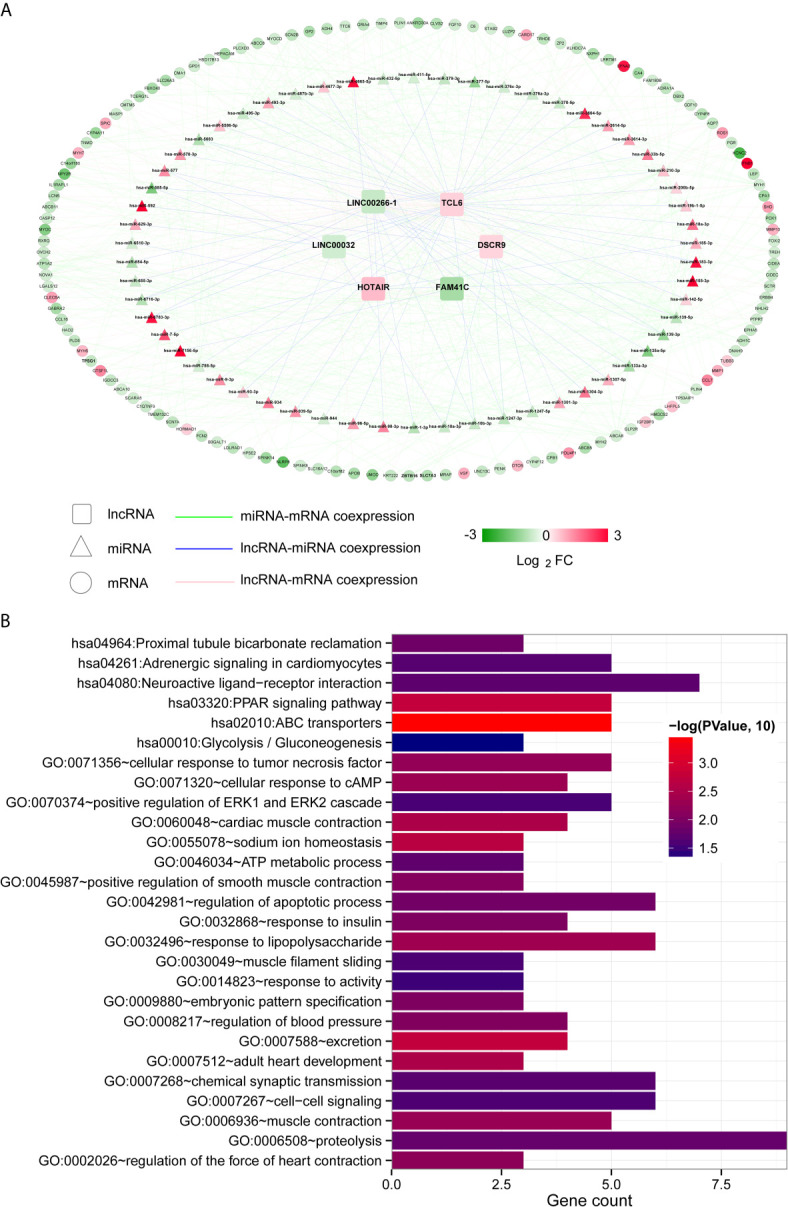
Coexpression ceRNA regulatory network. **(A)** The coexpression relationships among lncRNAs, miRNAs and mRNAs. The colour change from green to red indicates a change from low to high. **(B)** Bar diagram of the GO biological processes and KEGG signalling pathways significantly correlated with mRNAs in the ceRNA network. The horizontal axis depicts the number of genes. The vertical axis shows the entry names. Entries closer to a red colour are more significant.

Then, we used the Cor function in R to calculate the PCCs between DElncRNAs and DEmiRNAs, DElncRNAs and DEmRNAs and DEmiRNAs and DEmRNAs and selected the significantly correlated pairs (PCC > 0.4, *P* < 0.05). Finally, we obtained 69 pairs between DElncRNAs and DEmiRNAs, 158 pairs between DElncRNAs and DEmRNAs and 369 pairs between DEmiRNAs and DEmRNAs (**Table S4**). The three coexpression relationships were integrated to construct a comprehensive coexpression ceRNA network (**Figure 3A**). Next, a total of 21 significantly related GO biological processes and 6 KEGG signalling pathways were identified by DAVID based on enrichment annotation analysis of mRNAs in the coexpression regulatory network (**Figure 3B**). The top five GO terms related to miRNAs in the coexpression ceRNA network were mainly enriched in proteolysis, regulation of apoptotic processes, responses to lipopolysaccharide, chemical synaptic transmission and cell-cell signalling. The KEGG pathways related to these mRNAs were mainly enriched in ABC transporters, the PPAR signalling pathway, proximal tubule bicarbonate reclamation, neuroactive ligand-receptor interaction, adrenergic signalling in cardiomyocytes and glycolysis/gluconeogenesis.

### Screening for DEGs Associated With Survival Prognosis

**Figure 4:**
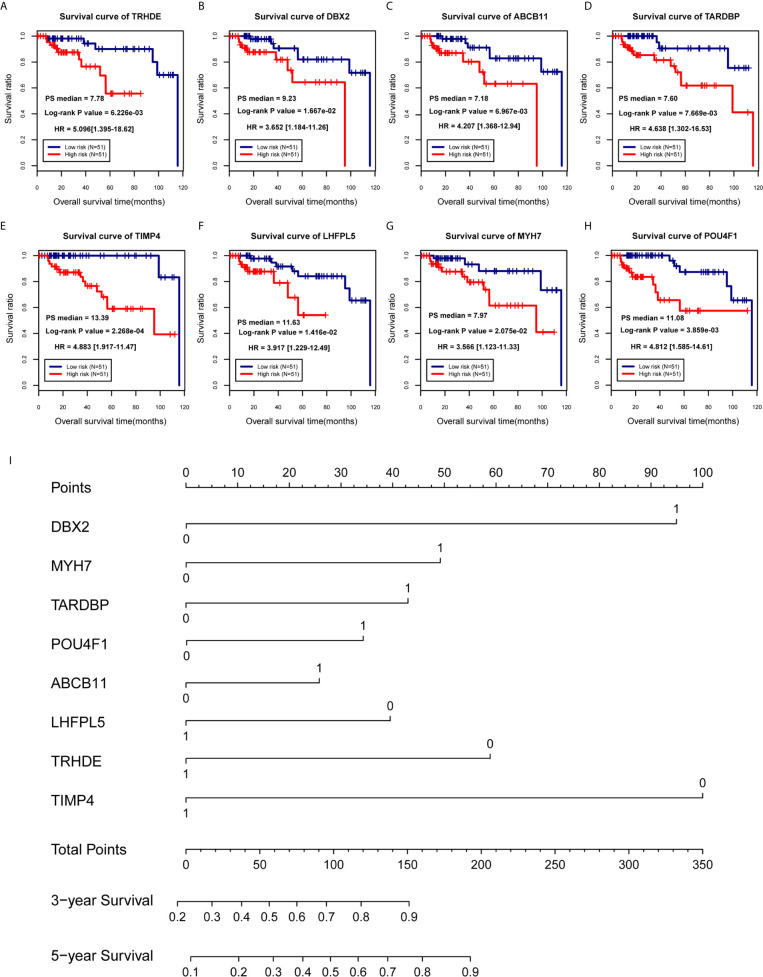
Establishment of a prognostic model for TNBC patients. **(A–H)** Survival curves of patients with TNBC, divided into low-risk and high-risk groups, carrying known prognostic genes. **(I)** Nomogram for the overall survival of TNBC patients.

According to 102 TNBC samples in the TCGA training set, a total of 144 mRNAs were identified based on the mRNAs contained in the two ceRNA regulatory networks. Then, 12 DEGs that were significantly associated with survival prognosis were identified by univariate Cox regression analysis (**Table S5**). Furthermore, multivariate Cox regression analysis was used to identify 8 DEGs associated with survival prognosis, including DBX2, MYH7, TARDBP, POU4F1, ABCB11, LHFPL5, TRHDE and TIMP4 (**Table 2**). Further, we found that ABCB11 involved in ABC transporters pathways and MYH7 involved in Adrenergic signaling in cardiomyocytes pathways. In GO, MYH7 participated in cardiac muscle contraction, POU4F1 participated in synapse assembly and neuron fate specification, TRHDE participated in regulation of blood pressure, proteolysis and cell-cell signaling, and TIMP4 participated in response to lipopolysaccharide. Then, we calculated the PS scores of the 8 DEGs and divided each gene into a high PS score group (PS score higher than or equal to the median PS score) and a low PS score group (PS score lower than the median PS score) according to the median PS score. Survival curves and log-rank tests were used to analyse the prognosis of each gene in patients (**Figures 4A–H**). The results showed that the PS scores of all 8 DEGs were significantly associated with survival prognosis. Therefore, a nomogram based on the prognostic factors that combined the 8 DEGs correlated with overall survival is presented in **Figure 4I**, indicating that the 8 DEGs was able to be an accurate predictor of survival in patients with TNBC.

**Table 2:** Univariate and multivariate analyses of independent prognostic mRNAs.

Symbol	Univariate analysis			Multivariate analysis			
	HR	95%CI	P value	Coefficient	HR	95%CI	P value
DBX2	0.899	0.807-0.962	2.650E-02	-0.340	0.712	0.553-0.916	8.120E-04
MYH7	0.825	0.728-0.934	1.250E-03	-0.214	0.807	0.676-0.963	1.739E-03
TARDBP	0.887	0.786-0.953	2.550E-02	-0.160	0.852	0.718-0.910	6.511E-03
POU4F1	0.903	0.805-0.941	4.200E-02	-0.147	0.864	0.729-0.923	8.915E-03
ABCB11	0.875	0.768-0.996	2.200E-02	-0.140	0.869	0.689-0.990	2.362E-02
LHFPL5	0.785	0.654-0.934	3.350E-03	0.183	1.200	1.151-1.516	1.245E-02
TRHDE	1.176	1.098-1.412	4.100E-02	0.282	1.326	1.124-1.901	1.258E-02
TIMP4	1.479	1.145-1.910	1.350E-03	0.457	1.579	1.188-2.808	1.196E-02

CI, confidence interval; HR, hazard ratio.

### Evaluation of Effectiveness and Comparison of the Prognostic Models

**Figure 5:**
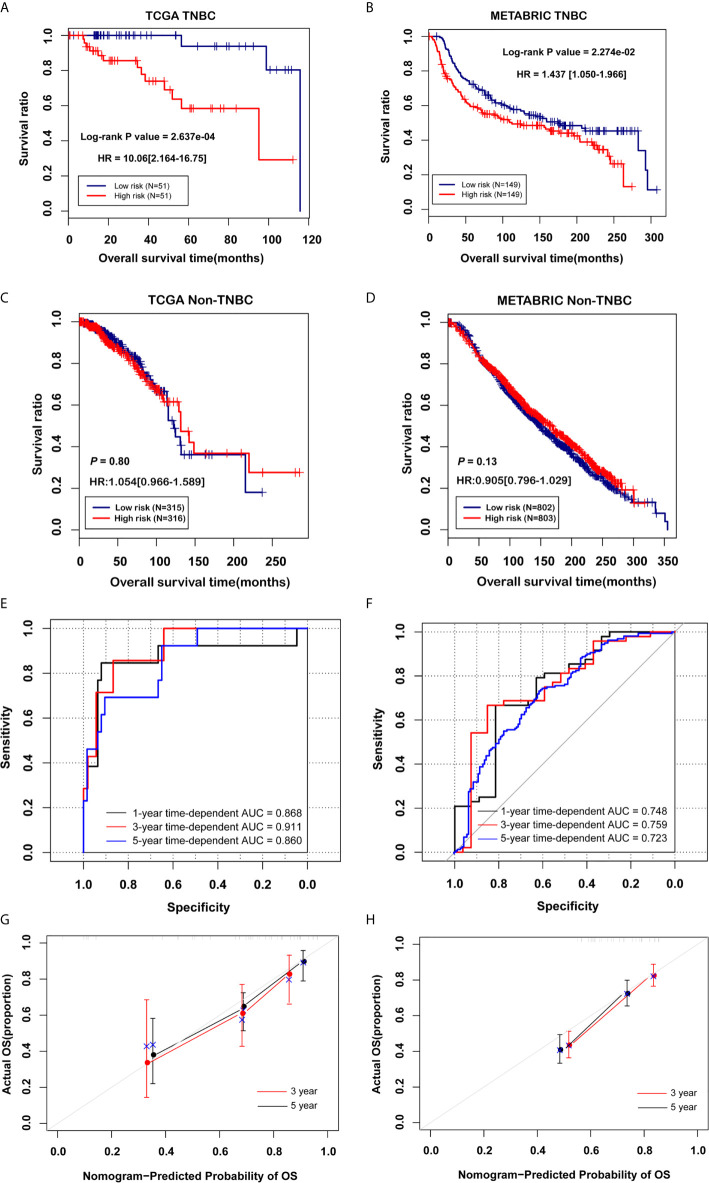
Validation of the prognostic model. **(A, B)** Kaplan-Meier curve based on the PS prediction model and prognostic correlation in TNBC groups. **(C, D)** Kaplan-Meier curve based on the PS prediction model and prognostic correlation in non-TNBC groups. **(E, F)** The ROC curve of the PS forecast model, with the black, red and blue curves representing the 1-year, 3-year and 5-year curves, respectively. **(G, H)** Three- and 5-year calibration curves based on the PS forecast models.

Based on the prognostic coefficients of the 8 DEGs from the multivariate Cox regression analysis and their expression levels in the TCGA training set obtained in the previous step, we constructed a prognostic score (PS) model and divided all samples from the TGCA training set into a high risk group (PS score equal to or higher than the PS median value) and a low risk group (PS score lower than the PS median value). Moreover, the corresponding mRNA expression levels were extracted from the Metabric validation dataset. Then, we calculated the PS score of each sample and divided them into a high-risk group and a low-risk group. The survival curve and log-rank test showed a predictability of the PS model groups for actual prognostic information for the disease (**Figures 5A, B** and **Table S6**). The survival rate of the low-risk group was significantly higher than that of the high-risk group in the TGCA training set (*P* < 0.01, HR = 10.06) and the Metabric validation set (*P* = 0.02, HR = 1.437). However, we test this PS model in non-TNBC cases and found that it was not associated with non-TNBC cases prognosis and did not work for non-TNBC (**Figures 5C, D**). Then, we applied ROC curves to assess the precision of the prognostic model. Similar to the nomogram, ROC curve analysis at 1, 3 and 5 years revealed area under the curve (AUC) values of 0.868, 0.911 and 0.860 in the TGCA training set and 0.748, 0.759 and 0.723 in the Metabric validation set, respectively (**Figures 5E, F**). In addition, the calibration curve showed that the nomogram-predicted and actual probability of survival at 3 and 5 years were in good agreement (**Figures 5G, H**). In brief, on the basis of PS model from 8 DEGs, we develop a predictive model for clinical application, which is accurate and effective for TNBC patients.

### Correlation of the Prognostic Model With Clinical Factors

**Figure 6:**
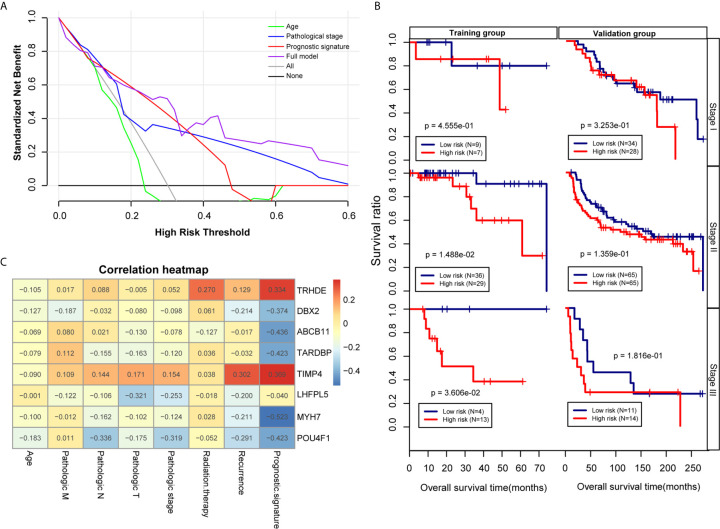
Effectiveness and comparison of the prognostic model. **(A)** Decision curve analysis. The green, blue, red and purple colours represent the decision curves for the patient’s age, pathological stage, PS model and combined model, respectively. The grey and black curves show the two extremes. **(B)** Kaplan-Meier curves were analysed by pathological stage stratification in the TCGA training set and Metabric validation set. **(C)** Heat map depicting the significance of correlations between clinical characteristics and 8 factors.

To further analysis the accuracy and application of the PS model compared with other clinical prognosis factors, multivariate Cox regression analyses were firstly carried out to evaluate the survival of TNBC patients using the prognostic model. The multivariate Cox regression analyses showed that pathological stage and prognostic model were independent prognostic factors in the TCGA training dataset. In the Metabric validation dataset, multivariate Cox regression analyses identified the patient’s age, pathologic stage and prognostic model as independent prognostic factors for overall survival (**Table 3**). Therefore, we performed decision curve analysis (DCA) of a single clinical factor, the PS model and clinical factors in combination with the PS model in the TCGA data set to compare the net effects of each variable on survival prognosis. As shown in **Figure 6A**, the prognostic model (red line) yielded a higher net profit than age (green line) and the pathological stage (blue line). DCA implied that the prognostic model combined with clinical factors was more beneficial that both a single clinical factor and the PS model by itself for forecasting the survival rate. Further subgroup analysis showed that the pathological stage was a significant factor influencing the prognosis of TNBC patients. As illustrated in **Figure 6B**, the survival rate in the high-risk group was obviously worse than that in the low-risk group, suggesting that the prognostic model was trustworthy and exhibited a steady predictive performance at distinct pathological subgroup stages. We performed correlation analyses of the 8 DEGs and the prognostic signature with TNBC clinical factors. The outcomes implied that the prognostic signature was considerably correlated with recurrence and the pathological stage (**Figure 6C** and **Table S7**).

**Table 3:** Univariate and multivariate analyses of the prognostic model.

Clinical characteristics	Univariate Cox			Multivariate Cox		
	HR	95%CI	P	HR	95%CI	P
**Training group (N=102)**						
Age (years, mean ± SD)	1.039	0.513-1.994	7.88E-01	1.004	0.964-1.045	8.62E-01
Pathological stage (I/II/III/IV/-)	4.465	2.197-9.824	**4.57E-05**	3.348	1.649-7.355	**1.07E-03**
Radiotherapy (Yes/No/-)	0.877	0.246-3.126	8.40E-01	0.833	0.507-2.730	3.77E-01
PS model status (High/Low)	10.06	2.164-16.75	**2.64E-04**	2.512	1.552-9.978	**1.75E-02**
**Validation group (N=298)**						
Age (years, mean ± SD)	1.024	1.012-1.036	**9.94E-05**	1.022	1.007-1.037	**3.31E-03**
Pathological stage (I/II/III/IV/-)	1.625	1.201-2.197	**1.57E-03**	1.687	1.249-2.278	**6.46E-04**
Radiotherapy (Yes/No/-)	0.841	0.601-1.177	3.13E-01	0.853	0.564-1.290	4.51E-01
PS model status (High/Low)	1.437	1.050-1.966	**2.27E-02**	1.541	1.071-2.219	**1.98E-02**

CI, confidence interval; HR, hazard ratio. The bold values represent statistically significant (P < 0.05).

### Construction of a Network for Small-Molecule Drugs and Characteristic Factors

**Figure 7:**
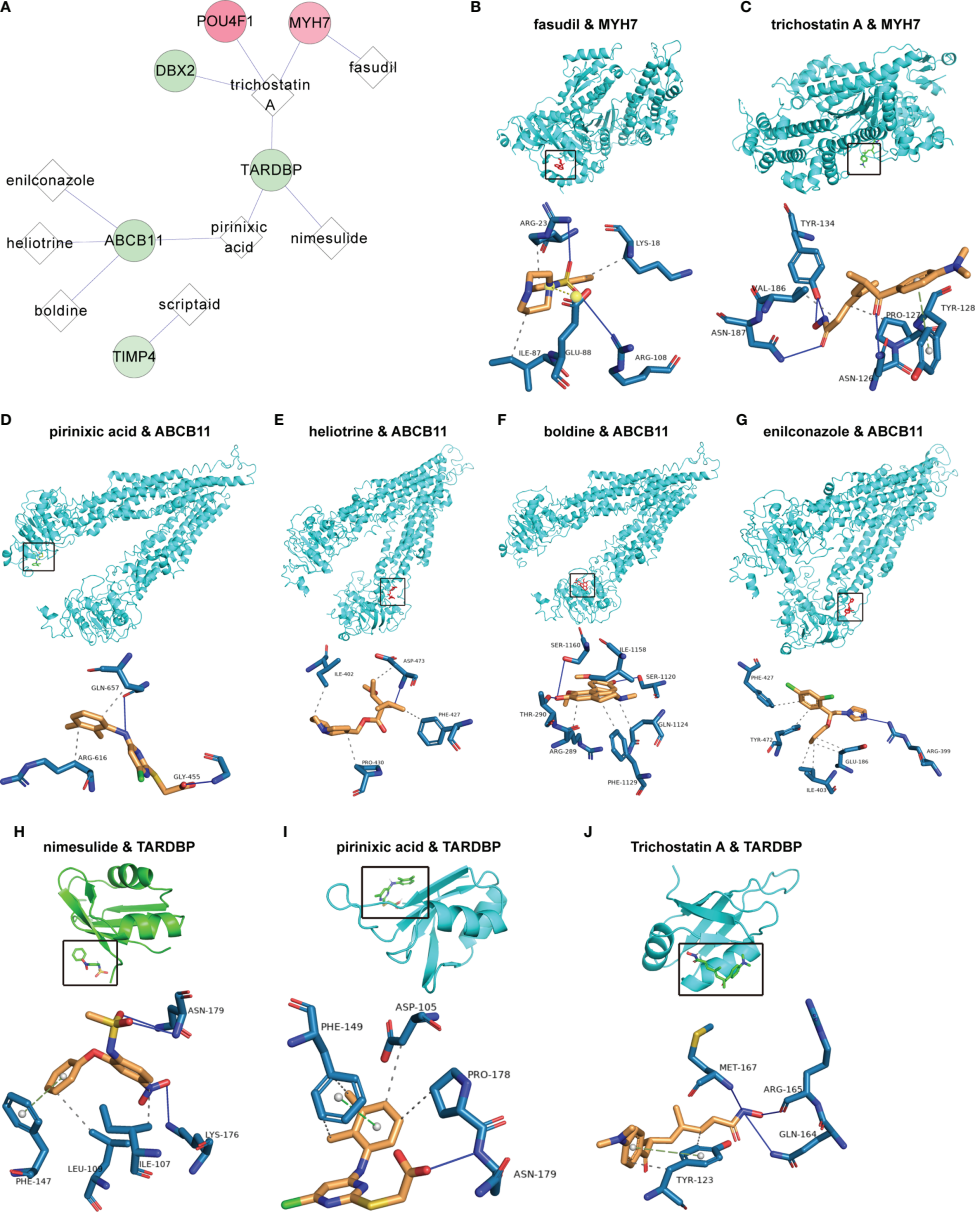
Correlation of the PS model characteristic features with small-molecule inhibitors. **(A)** Network of the associations of characteristic factors with small-molecule drugs. The circles and diamonds represent genes and drug molecules, respectively. **(B–J)** Molecular docking global diagram. Fasudil binding to MYH7 **(B)**, trichostatin A binding to MYH7 **(C)**, pirinixic acid binding to ABCB11 **(D)**, heliotrine binding to ABCB11 **(E)**, boldine binding to ABCB11 **(F)**, enilconazole binding to ABCB11 **(G)**, nimesulide binding to TARDBP **(H)**, pirinixic acid binding to TARDBP **(I)** and trichostatin A binding to TARDBP **(J)** are shown.

To explore the underlying treatment value of the PS model from 8 DEGs, we constructed the network of small-molecule drugs targeted 8 DEGs and predicted the possible binding sites. All the relationship data for genes and small-molecule drugs were downloaded from the CTD database, and 12 pairs were obtained from the 8 DEGs selected for construction of the PS model (**Figure 7A** and **Table S8**). Further, the docking possibilities between the target proteins and small molecules were assessed using AutoDock software (20). The results revealed 9 pairs from 3 DEGs and 8 small molecules (**Table 4**). Then, we analysed the local binding sites through PLIP online tools (https://projects.biotec.tu-dresden.de/plip-web/plip/index) and drew 3D structure simulations using PyMOL (version 2.4) (**Figures 7B–J**). According to the above analysis, the combination of fasudil and MYH7 was the best based on the affinity value (-8.2). Moreover, our results also provide a reference for developing clinical drugs according to characteristic factors.

**Table 4:** List of the best molecular docking model scores.

Rank	Molecule	Gene	Affinity
1	fasudil	MYH7	-8.2
2	trichostatin A	MYH7	-8
3	boldine	ABCB11	-6.8
4	pirinixic acid	ABCB11	-6.5
5	enilconazole	ABCB11	-6
6	heliotrine	ABCB11	-5.8
7	trichostatin A	TARDBP	-5.6
8	pirinixic acid	TARDBP	-5.1
9	nimesulide	TARDBP	-4.6

An affinity less than -5 indicates that the docking result was more reliable.

## Discussion

Noncoding RNAs (ncRNAs), which account for the majority (98%) of the transcriptome, are defined as gene transcripts with important biological functions (21). Among them, lncRNAs are one of the most important challenges biologists face today and represent potential new biomarkers and drug targets. The hypothesis of Salmena et al. (22) regarding ceRNA networks of multiple molecules involved in posttranscriptional regulation has received extensive attention in recent years. Their research showed that lncRNAs can act as ceRNAs to inhibit the function of miRNAs and communicate with mRNAs by carrying one or more miRNA response elements (MREs). However, the molecular biological mechanisms and effective regulatory networks affecting the progression and prognosis of TNBC remain unclear. It is important and helpful to optimize individualized treatments by exploring the molecular biological regulatory network and prognostic biomarkers of TNBC.

In this work, we used bioinformatic analyses to establish a new ceRNA regulatory network in TNBC that was based on 6 lncRNAs, 295 miRNAs, and 573 mRNAs. The ceRNA regulatory network was constructed in two ways: targeted regulation and coexpression regulation. A total of 144 mRNA nodes were found in the network. Among them, 12 mRNAs were significantly related to survival prognosis as determined by univariate Cox regression analysis, and an 8-mRNA prognostic nomogram was finally obtained *via* further multivariate Cox regression analysis. The 8-mRNA prognostic nomogram was helpful for identifying TNBC patients with a low survival probability. The OS rate in the high-risk group was significantly worse than that in the low-risk group in both the model and validation groups.

Many studies have explored prognostic biomarkers and possible molecular regulatory pathways in breast cancer, but studies on ncRNA-related cancers are commonly concentrated on miRNAs, lncRNAs and circRNAs (23–25). A corresponding lncRNA-miRNA-mRNA prognostic model of OS in TNBC patients has not been constructed. Jiang et al. (26) developed an integrated mRNA-lncRNA signature that effectively classifies TNBC patients into groups at low and high risk of disease recurrence, but the model is susceptible to the inherent biases of the study format and to the biases of the individuals performing the diagnoses and laboratory experiments. Therefore, we identified prognostic mRNA biomarkers and established a prognostic model based on a TCGA data set, while external verification was carried out using an independent external verification data set from the Metabric database. In this study, 8 potential biomarkers were found to be closely related to the OS of TNBC patients. Through the ceRNA regulatory network, potential lncRNA-miRNA-mRNA regulatory pathways are shown, which may help to clarify the potential biological mechanisms and regulatory pathways related to the OS of patients with TNBC. GO and KEGG pathway enrichments were further assessed to determine the potential molecular mechanism underlying the OS of TNBC patients.

Changes in TIMP4 expression have been reported to contribute to the development of breast cancer (27). In fact, TIMP4 is involved in several processes, including cell invasion and migration, cell proliferation and apoptosis, and angiogenesis (28). It can regulate the proteolytic activity of MMPS and is associated with ECM remodelling, EMT progression, and cancer initiation and progression by directly affecting cellular adhesion (29, 30). Different studies have associated high serum levels of MMPS and TIMPS with poor prognosis (31), and these factors were specifically identified as predictors of adverse outcomes in patients with breast cancer (32, 33). POU4F1 is a member of the POU domain family of transcription factors and plays a key role in regulating cancers. Studies have shown that POU4F1 expression is dramatically increased in breast cancer cells. POU4F1 deletion substantially downregulated the MEK1/2 and ERK1/2 signalling pathways in cancer cells (34, 35). TARDBP was originally considered to be an RNA/DNA binding protein and a regulator of HIV-1 gene expression. Increasing evidence shows that TARDBP may be involved in cell division, apoptosis and microRNA (miRNA) biogenesis (36). It is considered a powerful prognostic indicator of survival in breast cancer (37). In addition, ABCB11 mutations are prevalent in the DNA of patients with primary breast cancers and are considered to be associated with tumour prognosis (38). The results of all of these studies are compatible with the biomarker predictions in our study.

There are a few reports on the effects of other factors, such as DBX2, MYH7, TRHDE and LHFPL5, on TNBC. However, these factors are mainly studied in the context of other cancers. DBX2, a hypermethylated gene in the ctDNA of HCC patients, was identified as a potential biomarker and shows great promise for liquid biopsy applications in the future (39). It is significantly upregulated in HCC tissues and plays significant roles in the proliferation and metastasis of HCC cells by activating the Shh pathway (40). MHY7 mutations may play an important role in EBV-associated intrahepatic cholangiocarcinoma (41). TRHDE is reported to be a DNA methylation marker of oral precancer progression (42). Overexpression of the long noncoding RNA TRHDE-AS1 was shown to inhibit the progression of lung cancer *via* the miRNA-103/KLF4 axis (43). LHFPL5 is a member of the lipoma HMGIC fusion partner (LHFP) gene family and can cause deafness in humans. It is proposed to function in hair bundle morphogenesis. Studies have indicated that these biomarkers play important roles in the occurrence and development of tumours. In addition, our study found that some drugs could target these prognostic biomarkers by AutoDock software, including MYH7 targeted by fasudil and trichostatin A, ABCB11 targeted by boldine, pirinixic acid, enilconazole and heliotrine, and TARDBP targeted by trichostatin A, pirinixic acid and nimesulide. Previous studies showed that fasudil, as a ROCK inhibitor, could control tissue mechanics *via* regulation of Capzb in liver homeostasis (44). Trichostatin A is A histone deacetylase inhibitor with anti-breast cancer activity (45). Besides, Bodine has hepatoprotective, cell-protective, antipyretic and anti-inflammatory effects by antagonizing 5-HT3 receptors, and also against glioma cell lines (46, 47). Pirinixic acid inhibit 5- lipoxygenase activity and reduce vascular remodelling in the cardiovascular system (48). Some researches also found that enilconazole inhibited metastatic colorectal cancer through inhibition of TGF-β (49). What is more, nimesulide inhibited lung tumor growth through selective cyclocxygenase-2 (50). Therefore, it is necessary to perform experimental studies to elucidate the relevant pathogenesis and biological regulatory pathways in TNBC. Further studies need to be performed.

The current research has the following advantages. First, the prognostic nomograms provide a convenient way to estimate mortality at different time points. Second, a free network calculator of TNBC patient OS was developed and maintained to facilitate the assessment of individual mortality. Finally, as a noninvasive predictive tool, predictive nomograms provide an alternative for TNBC patients who cannot tolerate or do not want to undergo surgery.

Additionally, this study does have some limitations. First, the current study used gene expression data from the gene chip detection platform and lacks basic experimental verification. Second, the research data were generated on testing platforms, thereby affecting the repeatability of the results in different populations. Third, the model and validation data sets did not contain detailed research information, such as the drug treatment regimens and other postoperative treatments, which may affect the treatment efficacy and clinical prognosis.

Briefly, our research revealed potential molecular biological regulatory pathways and prognostic biomarkers through a ceRNA regulatory network. In particular, our prognostic model based on mRNAs in the ceRNA network showed a substantial ability to improve the prediction of OS in TNBC patients.

## Data Availability Statement

The original contributions presented in the study are included in the article/**Supplementary Material**. Further inquiries can be directed to the corresponding authors.

## Ethics Statement

Ethical approval was not required because researchers are allowed to download and analyse data from these databases for scientific purposes.

## Author Contributions

FQ, Y-SZ, PL and WQ designed the study, analysed the data and wrote the manuscript. FQ and JL collected the follow-up information and performed the clinical data analysis. FQ, JL, and WQ performed the bioinformatics analysis. All authors contributed to the article and approved the submitted version.

## Funding

This study was supported by the National Natural Science Foundation of China (grant nos. 81772590 and 81572395).

## Conflict of Interest

The authors declare that the research was conducted in the absence of any commercial or financial relationships that could be construed as a potential conflict of interest.

## Supplementary Material

Supplementary Table 1 Clinical information of the samples obtained from the TCGA and Metabric databases.

Supplementary Table 2 DERs obtained from meeting the threshold conditions.

Supplementary Table 3 Connection relationship of targeted ceRNA regulatory network.

Supplementary Table 4 Connection relationship of coexpression ceRNA regulatory network.

Supplementary Table 5 mRNAs in ceRNA network associated with survival prognosis.

Supplementary Table 6 PS score and grouping in training data set and verification data set.

Supplementary Table 7 Correlation coefficient and *p* value between clinical characteristics and 8 factors.

Supplementary Table 8 Correlation of the PS model characteristic features with small-molecule inhibitors from CTD database.

## Abbreviations

TCGA, The Cancer Genome Atlas; GEO, Gene Expression Omnibus; OS, overall survival; ROC-curve, Receiver operating characteristic curve; TNBC, triple-negative breast cancer; lncRNAs, long noncoding RNAs; DERs, differentially expressed RNAs; ceRNA, competitive endogenous RNA.
